# Supplementary material for: Inpatient morbidity and mortality of measles in the United States
Source: PLoS One. 2020 Apr 28;15(4):e0231329. doi: 10.1371/journal.pone.0231329 (PMC7188204; doi:10.1371/journal.pone.0231329)
Supplement: S1 Table — (DOCX) [file pone.0231329.s001.docx]

| **S1 Table. ICD-9-CM and ICD-10-CM Codes Used to Identify Measles and Complications** | |
| --- | --- |
| **Disorder** | **ICD-9-CM and ICD-10-CM Diagnosis Codes** |
| Measles | postmeasles encephalitis (055.0; B05.0), postmeasles pneumonia (055.1; B05.2), postmeasles otitis media (055.2; B05.3), measles keratoconjunctivitis (055.71; B05.81), measles with other specified complications (055.79; B05.1, B05.4), measles with unspecified complications (055.8; B05.89), measles without mention of complications (055.9; B05.9). |
| Dehydration | 276.5X; E86.0, E86.1, E86.9 |
| Diarrhea | 787.91; R19.7 |
| Enterocolitis | 558.9, K52.XXX |
| Hepatitis | 573.1, 573.2, 573.3; K72.XX, K75.XX, K76.XX, K77 |
| Nausea/Vomiting | 787.0X; R11.1X |
| Pancreatitis | 577.0, 577.1; K85.XX, K86.XX |
| Pancytopenia | 284.XX; D60.X, D61.XX |
| Thrombocytopenia | 283.XX; D694.X, D695.X |
| Cellulitis | 681.XX, 682.X; L03.XXX |
| Fever | 780.6X, R50.XX |
| Sepsis/SIRS | 995.XX; R65.XX |
| Septicemia | 038.XX; A41.XX |
| Encephalitis | 323.XX; G04.XX, G05.X |
| Meningitis | 320.XX, 321.X, 322.X; A87.X, A88.X, A89, G00.XX, G01, G02, G03.XX |
| Conjunctivitis | 372.0X, 372.1X, 372.2X, 372.3X; H10.XXX, H11.XXX |
| Keratitis | 370.XX; H16.XXX |
| Bronchitis | 466.XX; J20.X, J21.X, J22 |
| Otitis Media | 381.XX, 381.3, 381.4, 382.XX; H65.XXX, H66.XXX |
| Pleurisy | 511.XX; J94.X |
| Pneumonia | 480.X, 481, 482.XX, 483.X, 485, 486; J09.XX, J10.XX, J11.XX, J12.X, J13, J14, J15.X, J16.X, J17, J18.X |
| Acute Renal Failure | 584.X; N17.X |
| Hypocalcemia | 275.41; E83.51 |
| Hyposmolality / Hyponatremia | 276.1; E87.1 |
| Urinary Tract Infection | 599.0; N39.0 |
