# Supplementary material for: Inpatient morbidity and mortality of measles in the United States
Source: PLoS One. 2020 Apr 28;15(4):e0231329. doi: 10.1371/journal.pone.0231329 (PMC7188204; doi:10.1371/journal.pone.0231329)
Supplement: S2 Table — (DOCX) [file pone.0231329.s002.docx]

| **S2 Table. Predictors of Length of Stay in Patients with Measles** | | | | | | |
| --- | --- | --- | --- | --- | --- | --- |
| **Variable** | **No Measles** | | | **Measles** | | |
|  | **Least Squares Mean** | **Adjusted Beta**  **[95% CI]** | **P-value** | **Least Squares**  **Mean** | **Adjusted Beta**  **[95% CI]** | **P-value** |
| **Age** |  |  |  |  |  |  |
| 0-19 | 1.57 | 0 [ref] | - | 1.59 | 0 [ref] | - |
| ≥20 | 1.56 | -0.01 [-0.01, -0.01] | <0.0001 | 1.32 | -0.27 [-0.59, 0.04] | 0.089 |
| **Chronic Conditions** |  |  |  |  |  |  |
| 0-1 | 1.24 | 0 [ref] | - | 1.07 | 0 [ref] | - |
| ≥2 | 1.55 | 0.31 [0.31, 0.31] | <0.0001 | 1.83 | **0.76 [0.46, 1.06]** | **<0.0001** |
| **Discharge Quarter** |  |  |  |  |  |  |
| Jan-Mar | 1.58 | 0 [ref] | - | 1.46 | 0 [ref] | - |
| Apr-Jun | 1.56 | -0.02 [-0.02, -0.02] | <0.0001 | 1.39 | -0.06 [-0.45, 0.32] | 0.962 |
| Jul-Sep | 1.56 | -0.02 [-0.02, -0.02] | <0.0001 | 1.42 | -0.03 [-0.44, 0.37] | 0.994 |
| Oct-Dec | 1.56 | -0.01 [-0.02, -0.01] | <0.0001 | 1.55 | 0.09 [-0.36, 0.54] | 0.935 |
| **Hospital Location** |  |  |  |  |  |  |
| Metropolitan ≥1 Million | 1.59 | 0 [ref] | - | 1.71 | 0 [ref] | - |
| Metropolitan <1 Million / Micropolitan | 1.57 | -0.02 [-0.02, -0.02] | <0.0001 | 1.47 | -0.24 [-0.57, 0.09] | 0.194 |
| Not Metropolitan or Micropolitan | 1.54 | -0.05 [-0.05, -0.05] | <0.0001 | 1.18 | -0.53 [-1.30, 0.23] | 0.220 |
| **Income Quartile:** |  |  |  |  |  |  |
| 1^st^–3^rd^ | 1.57 | 0 [ref] | - | 1.51 | 0 [ref] | - |
| 4^th^ | 1.56 | 0.01 [0.01, 0.01] | <0.0001 | 1.40 | 0.11 [-0.19, 0.40] | 0.467 |
| **Primary Payer** |  |  |  |  |  |  |
| Medicare | 1.66 | 0 [ref] | - | 1.38 | 0 [ref] | - |
| Medicaid | 1.60 | -0.06 [-0.06, -0.06] | <0.0001 | 1.36 | -0.03 [-0.59, 0.54] | 0.999 |
| Private insurance | 1.50 | -0.16 [-0.16, -0.16] | <0.0001 | 1.32 | -0.06 [-0.53, 0.40] | 0.974 |
| Self-Pay / No Charge / Other | 1.50 | -0.16 [-0.16, -0.16] | <0.0001 | 1.75 | 0.37 [-0.18, 0.91] | 0.242 |
| **Race/Ethnicity** |  |  |  |  |  |  |
| White | 1.54 | 0 [ref] | - | 1.499 | 0 [ref] | - |
| Black | 1.60 | 0.06 [0.06, 0.06] | <0.0001 | 1.405 | -0.09 [-0.59, 0.40] | 0.951 |
| Hispanic | 1.55 | 0.01 [0.01, 0.01] | <0.0001 | 1.488 | -0.01 [-0.48, 0.46] | 0.999 |
| Asian/Pacific Islander/  Native American/Other | 1.57 | 0.03 [0.03, 0.03] | <0.0001 | 1.424 | -0.07 [-0.52, 0.37] | 0.965 |
| **Sex** |  |  |  |  |  |  |
| Male | 1.57 | 0 [ref] | - | 1.38 | 0 [ref] | - |
| Female | 1.56 | 0.02 [0.01, 0.02] | <0.0001 | 1.53 | -0.16 [-0.40, 0.09] | 0.208 |
| **Year** |  |  |  |  |  |  |
| 2002-2004 | 1.60 | 0 [ref] | - | 1.54 | 0 [ref] | - |
| 2005-2007 | 1.58 | -0.02 [-0.02, -0.02] | <0.0001 | 1.42 | -0.11 [-0.73, 0.51] | 0.958 |
| 2008-2010 | 1.56 | -0.04 [-0.04, -0.04] | <0.0001 | 1.65 | 0.11 [-0.50, 0.72] | 0.960 |
| 2011-2013 | 1.54 | -0.06 [-0.06, -0.05] | <0.0001 | 1.52 | -0.02 [-0.60, 0.57] | 0.999 |
| 2014-2016 | 1.55 | -0.05 [-0.05, -0.05] | <0.0001 | 1.14 | -0.40 [-0.98, 0.19] | 0.251 |
