# Supplementary material for: Inpatient morbidity and mortality of measles in the United States
Source: PLoS One. 2020 Apr 28;15(4):e0231329. doi: 10.1371/journal.pone.0231329 (PMC7188204; doi:10.1371/journal.pone.0231329)
Supplement: S3 Table — (DOCX) [file pone.0231329.s003.docx]

| **S3 Table. Predictors of Cost of Care in Patients with Measles** | | | | | | |
| --- | --- | --- | --- | --- | --- | --- |
| **Variable** | **No Measles** | | | **Measles** | | |
|  | **Least Squares**  **Mean** | **Adjusted Beta**  **[95% CI]** | **P-value** | **Least Squares**  **Mean** | **Adjusted Beta [95% CI]** | **P-value** |
| **Age** |  |  |  |  |  |  |
| 0-19 | 8.19 | 0 [ref] | - | 8.28 | 0 [ref] | - |
| ≥20 | 8.71 | 0.52 [0.52, 0.52] | <0.0001 | 8.11 | -0.16 [-0.56, 0.24] | <0.0001 |
| **Chronic Conditions** |  |  |  |  |  |  |
| 0-1 | 8.27 | 0 [ref] | - | 7.83 | 0 [ref] | - |
| ≥2 | 8.63 | 0.35 [0.35, 0.35] | <0.0001 | **8.56** | **0.73 [0.34, 1.12]** | **0.0003** |
| **Discharge Quarter** |  |  |  |  |  |  |
| Jan-Mar | 8.43 | 0 [ref] | - | 8.16 | 0 [ref] | - |
| Apr-Jun | 8.44 | 0.01 [0.01, 0.01] | <0.0001 | 8.11 | -0.05 [-0.53, 0.44] | 0.993 |
| Jul-Sep | 8.45 | 0.02 [0.02, 0.02] | <0.0001 | 8.20 | 0.05 [-0.47, 0.56] | 0.994 |
| Oct-Dec | 8.48 | 0.04 [0.04, 0.04] | <0.0001 | 8.31 | 0.16 [-0.41, 0.73] | 0.850 |
| **Hospital Location** |  |  |  |  |  |  |
| Metropolitan ≥1 Million | 8.50 | 0 [ref] | - | 8.67 | 0 [ref] | - |
| Metropolitan <1 Million / Micropolitan | 8.44 | -0.06 [-0.06, -0.06] | <0.0001 | 8.36 | -0.31 [-0.73, 0.11] | 0.186 |
| Not Metropolitan or Micropolitan | 8.40 | -0.10 [-0.10, -0.10] | <0.0001 | **7.56** | **-1.11 [-2.07, -0.15]** | **0.021** |
| **Income Quartile:** |  |  |  |  |  |  |
| 1^st^–3^rd^ | 8.39 | 0 [ref] | - | 8.28 | 0 [ref] | - |
| 4^th^ | 8.51 | -0.13 [-0.13, -0.13] | <0.0001 | 8.12 | 0.16 [-0.21, 0.53] | 0.390 |
| **Length of Stay** |  |  |  |  |  |  |
| 0-1 | 8.07 | 0 [ref] | - | 7.59 | 0 [ref] | - |
| 2 | 8.27 | 0.21 [0.21, 0.21] | <0.0001 | **8.17** | **0.58 [0.01, 1.14]** | **0.046** |
| ≥3 | 9.01 | 0.95 [0.95, 0.95] | <0.0001 | **8.82** | **1.23 [0.72, 1.74]** | **<0.0001** |
| **Primary Payer** |  |  |  |  |  |  |
| Medicare | 8.47 | 0 [ref] | - | 8.180883 | 0 [ref] | - |
| Medicaid | 8.40 | -0.07 [-0.07, -0.07] | <0.0001 | 8.158333 | -0.02 [-0.73, 0.68] | 0.999 |
| Private insurance | 8.54 | 0.07 [0.07, 0.07] | <0.0001 | 8.127448 | -0.05 [-0.63, 0.52] | 0.991 |
| Self-Pay / No Charge / Other | 8.39 | -0.08 [-0.08, -0.08] | <0.0001 | 8.314853 | 0.13 [-0.55, 0.82] | 0.924 |
| **Race/Ethnicity** |  |  |  |  |  |  |
| White | 8.42 | 0 [ref] | - | 8.304595 | 0 [ref] | - |
| Black | 8.37 | -0.04 [-0.05, -0.04] | <0.0001 | 8.036234 | -0.27 [-0.89, 0.36] | 0.629 |
| Hispanic | 8.50 | 0.09 [0.08, 0.09] | <0.0001 | 8.201452 | -0.10 [-0.70, 0.50] | 0.961 |
| Asian/Pacific Islander/Native American/Other | 8.51 | 0.10 [0.09, 0.10] | <0.0001 | 8.239234 | -0.07, [-0.65, 0.52] | 0.989 |
| **Sex** |  |  |  |  |  |  |
| Male | 8.50 | 0 [ref] | - | 8.26 | 0 [ref] | - |
| Female | 8.40 | 0.10 [0.10, 0.10] | <0.0001 | 8.13 | 0.13 [-0.18, 0.44] | 0.407 |
| **Year** |  |  |  |  |  |  |
| 2002-2004 | 8.44 | 0 [ref] | - | 8.03 | 0 [ref] | - |
| 2005-2007 | 8.50 | 0.07 [0.07, 0.07] | <0.0001 | 8.37 | 0.34 [-0.48, 1.15] | 0.611 |
| 2008-2010 | 8.53 | 0.09 [0.09, 0.10] | <0.0001 | 8.28 | 0.25 [-0.54, 1.03] | 0.783 |
| 2011-2013 | 8.43 | -0.01 [-0.01, -0.01] | <0.0001 | 8.23 | 0.20 [-0.56, 0.96] | 0.861 |
| 2014-2016 | 8.35 | -0.09 [-0.09, -0.09] | <0.0001 | 8.06 | 0.03 [-0.75, 0.80] | 0.999 |
