# Supplementary material for: Inpatient morbidity and mortality of measles in the United States
Source: PLoS One. 2020 Apr 28;15(4):e0231329. doi: 10.1371/journal.pone.0231329 (PMC7188204; doi:10.1371/journal.pone.0231329)
Supplement: Attachment — Submitted filename: REVIEWERS COMMENT PLOSONE JANUARY 2020.docx [file pone.0231329.s004.docx]

REVIEWERS COMMENTS PLOSONE JANUARY 2020

(PONE-D-19-307-45)

Reviewer’s Report

Title: Inpatient Morbidity and Mortality of Measles in the United States

Date: 11.01.2020

Version 1

**General comments:** Topic is of relevance and global interest. Reads well but introduction and methodology do not contain sufficient detail to appreciate study and anticipate results.

Title: Title seems appropriate. Suggest adding source of data (discretionary)

**Abstract:**

Page 3 line 48-49, 52-53: A concise summary but more information on background and analysis would be useful.

Page 3: Line 62; suggest writing defining vs by also writing it in full and defining other abbreviations when first mentioned.

**Introduction:**

Page 4 line 84: suggest removing “that”

**Introduction**

Suggest adding additional information such as - Information on the epidemiology of the most recent outbreaks in the US, any changes in predictors of hospitalisation in the US over the years or experience from other developed countries - useful for understanding of the context surrounding this study and changes to expect.

**Methods:**

**Study area: No** detail. It would be useful to know the geographic distribution of hospitals included in the sample, rural -urban differences etc whether it cover high risk locations e.g. migrant populations?

Page 6 line 97- Sampling: Suggest more detail is provided on the sampling procedure – what criteria informs the weighting? How many hospitals were represented in this sample? What types of hospitals were included, how many were children’s hospital’s?

Did the sample include hospitals from communities that had an outbreak during the period and were these populations targeted?

**Study Population:** What were the inclusion criteria- ICD 9/10 criteria alone? What proportion had laboratory confirmation? The age of patients was not specified? Were there any other exclusion criteria?

**Data collection**

Page 6: line 103- 10: What information was collected from the database? Though some is reported with the statistical analysis, but this aspect is not clear. It will be helpful to know the information collected on patient level characteristics, hospital level characteristics and outcome measures? Was there collection of data on immunisation status?

Page 6 line 106-7: Provide more detail on the co-morbidities studied especially those known to be associated with hospitalisations from measles what proportion had chronic respiratory conditions? Was it a risk factor for hospitalisations and longer duration of stay? Was this analysis done?

**Results**

Page 9 line Table 1 what chronic conditions?

Page 10 line 153 – suggest “Factors associated with hospitalization for measles”

Page 10: line 164 – “complications” kindly rephrase and make it clearer .

Page 10 line 169 - any sex differences?

Page 13 line 192 and 217 also need to be rephrased.

Page13 line 208; Define abbreviation ED

Why were children <5 years not considered as a separate group in the analysis?

**Discussion**

Extensive and covers findings

Page 17 line 287: contains mortality data not seen in results

Limitation Page 19 lines 331 and 332 should have also been in the methods section.

**Conclusion**

Section for conclusion not highlighted and should relate to objectives and findings of study.

Graphs: some graphs do not have titles

References:

Page 29 line 366-369 in capitals – any reason?

Suggest including more recent articles.
