# Supplementary material for: Inpatient morbidity and mortality of measles in the United States
Source: PLoS One. 2020 Apr 28;15(4):e0231329. doi: 10.1371/journal.pone.0231329 (PMC7188204; doi:10.1371/journal.pone.0231329)
Supplement: Attachment — Submitted filename: Chovatiya_Measles_Paper_V10_PLOSOne (Cmnt ASB).docx [file pone.0231329.s005.docx]

**Title: Inpatient morbidity and mortality of measles in the United States**

**Short Title: Measles hospitalization**

**Raj Chovatiya, MD, PHD^1^, Jonathan I. Silverberg, MD, PhD, MPH^2*^**

1. Department of Dermatology, Northwestern University Feinberg School of Medicine, Chicago, IL 60611
2. Department of Dermatology, The George Washington University School of Medicine and Health Sciences, Washington, DC, 20037

*Corresponding author:

E-mail: JonathanISilverberg@Gmail.com (JIS)

**Word count:** 3,236

**Abstract word count:** 192

**Figures:** 2

**Tables:** 2

**Supplemental Tables:** 3

**Key Words:** measles; vaccination; outbreak; mortality; hospitalization; burden; cost of care; length of stay; comorbidities

**Abbreviations used:** ICD-9-CM = International Classification of Disease 9^th^ edition Clinical Modification; ICD-10-CM = International Classification of Disease 10^th^ edition Clinical Modification; NIS = Nationwide Inpatient Sample; HCUP=Healthcare Cost and Utilization Project; CDC = Centers of the Disease Control and Prevention

JI Silverberg had full access to all the data in the study and takes responsibility for the integrity of the data and accuracy of the data analysis.
Study concept and design: JI Silverberg
Acquisition of Data: R Chovatiya and JI Silverberg
Analysis and interpretation of data: JI Silverberg, R Chovatiya
Drafting of the manuscript: JI Silverberg, R Chovatiya
Critical revision of the manuscript for important intellectual content: JI Silverberg, R Chovatiya
Statistical analysis: R Chovatiya and JI Silverberg
Obtained funding: JI Silverberg and R Chovatiya
Administrative technical or material support: None
Study supervision: None
Financial disclosures: None
Funding Support: This publication was made possible with support from the Agency for Healthcare Research and Quality (AHRQ) (JIS), the Dermatology Foundation (JIS), NIH K12 HS023011 (JIS), and NIH T32 AR060710 (RC)

Design and conduct of the study? No

Collection, management, analysis and interpretation of data? No

Preparation, review, or approval of the manuscript? No

Decision to submit the manuscript for publication? No

Conflicts of interest: None

**Abstract**

**Background:** Measles outbreaks are increasingly occurring in the US. Yet, little is known about hospitalization and complications from measles in recent years.

**Objectives:** To analyze the frequency, predictors, costs and other outcomes of hospitalization for measles in the US.

**Methods:** The 2002-2016 Nationwide Inpatient Sample, containing a 20% sample of US hospitalizations (n=96,568,625), was analyzed.

**Results:** Overall, 1,018 measles hospitalizations occurred in 2002-2016, and hospitalizations increased over time. In multivariable logistic regression models, measles was associated with higher odds of gastrointestinal, hematologic, infectious, neurologic, ophthalmologic, pulmonary, and renal complications, with the strongest association observed with encephalitis (39.84 [16.51-96.12], P<0.0001). Increased length of stay (LOS) and similar cost of care (mean [95% CI]; 4.8 [4.4-5.4]; $7,438 [$6,446-$8,582]) were observed vs. all other admissions (4.5 [4.4-4.5]; P<0.01; $7,854 [$7,774-$7,935], P>0.05). There were 34 deaths in hospitalized measles patients; inpatient mortality was numerically higher in those with vs. without measles (proportion ± SEM: 3.3±1.2% vs. 2.3±0.01%, P=0.333).

**Limitations:** Lack of outpatient or prescription data.

**Conclusions:** Measles continues to pose a substantial and preventable health care burden, with serious complications, hospitalization and inpatient mortality. Further studies are needed to improve the prevention and management of measles.

**Introduction**

Measles is a highly contagious and potentially life-threatening, airborne disease characterized by high fever, cough, coryza, conjunctivitis, and morbilliform rash. Measles can cause numerous organ-specific complications that may lead to inpatient hospitalization and even death, including gastrointestinal [1-3], neurologic [4-9], pulmonary [10-13], ophthalmologic [14-16], hematologic [17], renal [18], and dermatologic complications [19].

Prior to the introduction of measles vaccination in 1963, there were >100 million measles cases resulting in 6 million deaths worldwide, with 4 million cases and 450 deaths in the US annually [20]. Despite major strides in vaccine coverage, measles is still a leading cause of vaccine-preventable death, especially in children, with more than 20 million new cases and 100,000 deaths worldwide annually [21,22]. In the US, due to a highly successful public health campaign based on universal vaccination, measles was officially declared eliminated (i.e., absence of endemic transmission for ≥12 months) in 2000 [23,24]. However, the US has faced a resurgence of measles outbreaks in recent years, driven largely by travel-related exposures and communities with low rates of vaccination [25]. According to the Centers of the Disease Control and Prevention (CDC), there have already been 1,250 confirmed cases of measles in 31 states with 119 hospitalizations from January to October 2019, the highest yearly total since the year 1992; this trend that has been mirrored worldwide.

Severe cases of measles require hospitalization. Based on historical data, the CDC has estimated that approximately 1 in 4 of cases of measles in the US result in hospitalization, and 1 in 1000 cases results in death. Hospitalizations for measles precipitously declined with widespread measles vaccination [26,27]. Without vaccination, there would be 400,000 hospitalizations costing >$3 billion USD and >1,800 deaths annually [27,28]. However, few studies examined the occurrence of complications, hospitalization, and mortality secondary to measles since its resurgence in the US. This study sought to analyze the frequency, predictors, costs and other outcomes of hospitalization for measles in the US.

**Materials and methods**

The 2002–2016 Nationwide Inpatient Sample (NIS) was analyzed. The NIS is sponsored by the Healthcare Cost and Utilization Project (HCUP) of the Agency for Healthcare Research and Quality. Each year of the NIS contains an approximately 20% stratified representative sample of all hospitalizations in the US. Sample weights were created by the NIS that factored in the study sampling design allow for representative estimates of hospital discharges across the US. All data were de-identified, and no attempts were made to identify any of the individuals in the database. Patient consent was not obtained because the databases were received de-identified. All parties with access to the HCUP were compliant with the HCUP’s formal data use agreement. This study was approved by the institutional review board at Northwestern University.

The NIS lists one primary diagnosis and up to 24 secondary diagnoses. The databases were searched for a primary or secondary diagnosis of measles and comorbidities using International Classification of Disease, Ninth Revision, Clinical Modification (ICD-9-CM) or ICD-10-CM codes (S1 Table). Comorbid diagnoses were selected based on previous associations in the literature or to explore for novel associations.

To control for readmitted cases that may be counted as unique encounters in the NIS, we excluded patients who were transferred to short-term hospitals with planned readmission (4.8% of measles cases in the cohort).

**Data analysis**

Statistical analysis was performed using survey procedures adjusting for sample weighting, clustering, and strata in SAS version 9.4 (SAS Institute, Cary, NC). Baseline characteristics of inpatients with and without a measles diagnosis were determined. The cost for inpatient care was calculated based on the total charge of the hospitalization and the cost-to-charge ratio estimated by HCUP. Costs were adjusted for inflation to the year 2018 according to the Consumer Price Index from the US Bureau of Labor Statistics. Weighted t-tests and Rao-Scott chi-square tests compared the characteristics of mean and categorical variables, respectively.

Associations of measles hospitalization were examined including age, number of chronic conditions, discharge quarter, sex, health insurance coverage, hospital location, race/ethnicity, median annual income of the hospital zip code, and year. Multivariable logistic regression models were constructed with measles diagnosis as the independent variable (yes/no) and various complications (yes/no) as the dependent variables. Multivariable linear regression models were constructed with measles diagnosis as the independent variable (yes/no), and log-transformed cost or LOS as the dependent variables (continuous). Cost and LOS were log-transformed because model residuals were not normally distributed for the untransformed variables. Complete case-analysis was performed. Post-hoc correction for multiple dependent tests was performed by minimizing the false discovery rate with the approach of Benjamini and Hochberg [29]. Two-sided, corrected P-values ≤0.05 were considered statistically significant.

**Results**

**Population characteristics**

There were 96,568,625 discharges (weighted frequency: 466,712,770) analyzed in the NIS between 2002 and 2016, including 1,018 weighted cases of hospitalization for measles. The estimated incidence of measles hospitalizations was 2.2 per ten million persons [ptm] and increased between 2002 and 2016 (Fig 1A).

**Fig 1.** Measles hospitalizations by year (A), age stratified by ICD code (B), and ICD code stratified by age (C).

The mean ± standard error of the mean (SEM) age of measles inpatients was 32.0 ± 1.9 years, with a majority being <10 and ≥40 years (32.0% and 41.3%) and male (56.1%) (Table 1). Of the measles inpatients <10 years of age, 64.5% were 0-1 year, 25.1% were 2-5 years, and 10.4% were 6-9 years. The incidence of hospitalization decreased with age (0-9 years: 5.4 ptm; ≥40 years: 2.0 ptm) (Fig 1B). Most patients were healthy at baseline with ≤1 chronic comorbid condition (51.8%) and presented to hospital in a metropolitan area with population ≥ 1 million (62.9%). Private insurance (39.6%) and Medicaid (28.5%) were the most common payment sources. Inpatients with vs. without measles were significantly more likely to have non-white race/ethnicity overall (44.4% vs. 31.3%), including Hispanics (18.3% vs. 10.7%), and Asians/Pacific Islanders (9.0% vs. 2.3%). There were no significant differences in discharge season and income quartile in patients with vs. without measles.

| **Table 1. Baseline Characteristics of Inpatients With and Without Measles** | | | |
| --- | --- | --- | --- |
| **Variable** | **Measles** | | |
|  | **No** | **Yes** | **P-value** |
| **Age – mean (SEM)** | 54.6 (0.1) | 32.0 (1.9) | <0.0001 |
| **Age – wtd freq (%)** |  |  |  |
| 0-9 | 34,914,571 (7.5%) | 325 (32.0%) | <0.0001 |
| 10-19 | 14,718,915 (3.2%) | 53 (5.2%) |  |
| 20-39 | 71,217,494 (15.3%) | 219 (21.5%) |  |
| ≥40 | 345,466,299 (74.1%) | 421 (41.1%) |  |
| **Sex – wtd freq (%)** |  |  |  |
| Male | 208,835,843 (44.8%) | 571 (56.1%) | 0.001 |
| Female | 256,996,879 (55.2%) | 447 (43.9%) |  |
| **Chronic Conditions – wtd freq (%)** |  |  |  |
| 0-1 | 87,270,795 (20.7%) | 460 (51.8%) | <0.0001 |
| 2-5 | 185,504,649 (44.0%) | 296 (33.4%) |  |
| ≥6 | 149,220,170 (35.3%) | 132 (14.9%) |  |
| **Died – wtd freq (%)** |  |  |  |
| No | 455,483,562 (97.7%) | 984 (96.7%) | 0.333 |
| Yes | 107,88,277 (2.3%) | 34 (3.3%) |  |
| **Discharge Quarter – wtd freq (%)** |  |  |  |
| Jan-Mar | 119,003,074 (25.5%) | 271 (26.8%) | 0.379 |
| Apr-Jun | 116,383,379 (25.0%) | 294 (29.0%) |  |
| Jul-Sep | 115,217,670 (24.7%) | 220 (21.7%) |  |
| Oct-Dec | 115,644,962 (24.8%) | 228 (22.5%) |  |
| **Hospital Location – wtd freq (%)** |  |  |  |
| Metropolitan ≥ 1 Million | 230,781,973 (53.4%) | 593 (62.9%) | 0.021 |
| Metropolitan < 1 Million | 121,268,896 (28.0%) | 233 (24.8%) |  |
| Micropolitan | 47,133,884 (10.9%) | 82 (8.7%) |  |
| Not Metropolitan or Micropolitan | 33,157,800 (7.7%) | 34 (3.7%) |  |
| **Income quartile – wtd freq (%)** |  |  |  |
| 1^st^ | 127,041,374 (29.7%) | 243 (26.2%) | 0.472 |
| 2^nd^ | 111,244,640 (26.0%) | 247 (26.6%) |  |
| 3^rd^ | 101,120,717 (23.6%) | 208 (22.5%) |  |
| 4^th^ | 88,357,189 (20.7%) | 230 (24.8%) |  |
| **Primary Payer – wtd freq (%)** |  |  |  |
| Medicare | 209,457,726 (45.0%) | 179 (17.6%) | <0.0001 |
| Medicaid | 75,330,911 (16.2%) | 290 (28.5%) |  |
| Private insurance | 140,036,817 (30.1%) | 403 (39.6%) |  |
| Self-Pay / No Charge | 25,579,210 (5.5%) | 121 (11.9%) |  |
| Other | 15,433,769 (3.3%) | 24 (2.4%) |  |
| **Race/Ethnicity – wtd freq (%)** |  |  |  |
| White | 269,191,673 (68.7%) | 510 (55.6%) | <0.0001 |
| Black | 57,304,804 (14.6%) | 88 (9.6%) |  |
| Hispanic | 42,076,891 (10.7%) | 168 (18.3%) |  |
| Asian/Pacific Islander | 9,051,815 (2.3%) | 83 (9.0%) |  |
| Native American | 2,360,698 (0.6%) | ≤10 (0.6%) |  |
| Other | 12,095,380 (3.1%) | 63 (6.9%) |  |

**Associations with hospitalization for measles**

Hospitalization for measles was associated with male sex (survey logistic regression: adjusted OR [95% CI]: 1.48 [1.27-1.72]) compared with females, Medicaid (1.29 [1.06-1.55]) or no insurance (1.64 [1.31-2.06]) compared with private, and Asian/Pacific Islander (3.24 [2.45-4.29]), Hispanic (1.35 [1.10-1.67]), or Native American/other (2.12 [1.62-2.77]) race/ethnicity compared with whites. Demographic factors inversely associated with measles hospitalization included increasing age (≥20 years: 0.38 [0.32-0.46] compared to 0-19 years), increasing number of chronic comorbid conditions (≥2: 0.57 [0.48-0.69] compared to ≤1), and decreasing urban population (small metropolitan/micropolitan: 0.78 [0.67-0.92]; non-metropolitan/non-micropolitan: 0.51 [0.34-0.78] compared to metropolitan areas with ≥1 million population).

**Complications**

Across all ages, the majority of measles inpatients were diagnosed as measles without mention of complications (82.1%) (Fig 1C). Diagnoses of measles with various specified complications, including encephalitis, pneumonia, and keratoconjunctivitis, generally had higher prevalence with increasing age, while measles without mention of complications was diagnosed more commonly in younger age (Fig 1D).

The most frequent complications observed with measles were: dehydration (weighted frequency [%]: 161 [15.8%]), hyponatremia (145 [14.3%]), pneumonia (127 [12.5%]), acute renal failure (106 [10.4%]), diarrhea (97 [9.5%]), thrombocytopenia (97 [9.5%]), conjunctivitis (87 [8.5%]), septicemia (84 [8.3%]), fever (73 [7.2%]), sepsis/SIRS (64 [6.3%]), bronchitis (49 [4.8%]), pleurisy (38 [3.8%]), otitis media (37 [3.7%]), and pancytopenia (35 [3.4%]) (Table 2). Even among inpatients who were diagnosed as measles without mention of complications, 15.7% had dehydration, 14.5% had hyponatremia, 13.4% had pneumonia, 10.5% had diarrhea, 9.9% had thrombocytopenia, 9.3% had acute renal failure, 8.1% had fever, 8.1% had conjunctivitis, 7.6% had septicemia, and 5.2% had sepsis.

In multivariable survey logistic regression models including age, race/ethnicity, and sex as covariables, measles was associated with numerous organ-specific complications: gastrointestinal (aOR [95% CI] for dehydration: 3.96 [2.63-5.96]; diarrhea: 8.18 [5.12-13.05]; hepatitis: 4.37 [1.65-11.55]), hematologic (pancytopenia: 5.98 [2.64-13.57]; thrombocytopenia: 3.88 [2.32-6.51]), infectious (fever: 2.29 [1.13-4.65]; sepsis/SIRS: 2.73 [1.51-4.92]; septicemia 3.15 [1.88-5.28]), neurologic (encephalitis: 39.84 [16.51-96.12]; meningitis: 4.11 [1.32-12.80]), ophthalmologic (conjunctivitis: 27.20 [10.90-50.14]; keratitis: 34.86 [5.19-234.24]), pulmonary (otitis media: 3.17 [1.43-7.02]; pleurisy: 3.01 [1.41-6.44]; pneumonia of any cause: 2.27 [1.43-3.58]; bacterial pneumonia: 3.06 [1.73-5.42]; viral pneumonia: 3.83 [1.01-14.67]), and renal (acute renal failure: 2.65 [1.64-4.26]; hypocalcemia: 5.17 [2.15-12.41]; hyponatremia: 4.75 [2.59-8.72]). Enterocolitis (2.21 [1.02-4.82]) and bronchitis (4.33 [2.30-8.14]) showed higher odds only in bivariable models.

| **Table 2. Association of Measles with Organ-Specific Complications** | | | | | | |
| --- | --- | --- | --- | --- | --- | --- |
|  | **Measles** | | | | | |
|  | **No** | **Yes** | | | | |
| **Complications** | **Wtd Freq (%)** | **Wtd Freq (%)** | **Crude OR [95% CI]** | **P-value** | **Adjusted OR [95% CI]** | **P-value** |
| **Gastrointestinal** |  |  |  |  |  |  |
| Dehydration | 27,650,224 (5.9%) | 161 (15.8%) | **2.99 [2.06-4.35]** | **<0.0001** | **3.96 [2.63-5.96]** | **<0.0001** |
| Diarrhea | 7,937,366 (1.7%) | 97 (9.5%) | **6.12 [3.88-9.66]** | **<0.0001** | **8.18 [5.12-13.05]** | **<0.0001** |
| Enterocolitis | 6,125,651 (1.3%) | 28 (2.7%) | **2.21 [1.02-4.82]** | **0.045** | 2.15 [0.95-4.84] | 0.065 |
| Hepatitis | 2,483,470 (0.5%) | 24 (2.4%) | **4.61 [1.95-10.87]** | **0.001** | **4.37 [1.65-11.55]** | **0.003** |
| Nausea/Vomiting | 10,218,733 (2.2%) | 35 (3.5%) | 1.62 [0.76-3.43 | 0.210 | 1.75 [0.82-3.73] | 0.146 |
| Pancreatitis | 7,647,125 (1.6%) | ≤10 (0.5%) | 0.33 [0.06-1.89] | 0.211 | 0.34 [0.06-2.03] | 0.234 |
| **Hematologic** |  |  |  |  |  |  |
| Pancytopenia | 2,698,812 (0.6%) | 35 (3.4%) | **6.20 [2.94-13.09]** | **<0.0001** | **5.98 [2.64-13.57]** | **<0.0001** |
| Thrombocytopenia | 13,097,374 (2.8%) | 97 (9.5%) | **3.42 [2.11-5.54]** | **<0.0001** | **3.88 [2.32-6.51]** | **0.438** |
| **Infectious / Other** |  |  |  |  |  |  |
| Cellulitis | 19,082,604 (4.1%) | 42 (4.2%) | 1.57 [0.76-3.25] | 0.222 | 1.05 [0.52-2.15] | 0.886 |
| Fever | 4,802,035 (1.0%) | 73 (7.2%) | **7.68 [4.50-13.10]** | **<0.0001** | **2.29 [1.13-4.65]** | **0.022** |
| Sepsis/SIRS | 16,259,512 (3.5%) | 64 (6.3%) | **1.89 [1.09-3.31]** | **0.025** | **2.73 [1.51-4.92]** | **0.001** |
| Septicemia | 20,204,516 (4.3%) | 84 (8.3%) | **2.04 [1.25-3.33]** | **0.004** | **3.15 [1.88-5.28]** | **<0.0001** |
| **Neurologic** |  |  |  |  |  |  |
| Encephalitis | 250645 (0.1%) | 24 (2.4%) | **46.77 [19.63-111.44]** | **<0.0001** | **39.84 [16.51-96.12]** | **<0.0001** |
| Meningitis | 976,510 (0.2%) | 14 (1.3%) | **6.72 [2.25-20.11]** | **0.001** | **4.11 [1.32-12.80]** | **0.015** |
| **Ophthalmologic** |  |  |  |  |  |  |
| Conjunctivitis | 1,064,164 (0.2%) | 87 (8.5%) | **41.48 [26.03-66.08]** | **<0.0001** | **27.20 [10.90-50.14]** | **<0.0001** |
| Keratitis | 82,602 (0.02%) | ≤10 (0.5%) | **28.38 [4.107-196.10]** | **0.0007** | **34.86 [5.19-234.24]** | **0.0003** |
| **Pulmonary** |  |  |  |  |  |  |
| Bronchitis | 5,422,706 (1.2%) | 49 (4.8%) | **4.33 [2.30-8.14]** | **<0.0001** | 1.31 [0.49-3.55] | 0.594 |
| Otitis Media | 1,752,201 (0.4%) | 37 (3.7%) | **10.14 [5.07-20.28]** | **<0.0001** | **3.17 [1.43-7.02]** | **0.004** |
| Pleurisy | 9,186,882 (2.0%) | 38 (3.8%) | **2.01 [1.01-4.02]** | **0.048** | **3.01 [1.41-6.44]** | **0.004** |
| Pneumonia (all) | 37,095,861 (7.9%) | 127 (12.5%) | **1.66 [1.09-2.53]** | **0.017** | **2.27 [1.43-3.58]** | **0.001** |
| Pneumonia (bacterial) | 19,062,938 (4.1%) | 79 (7.8%) | **2.04 [1.24-3.36]** | **0.005** | **3.06 [1.73-5.42]** | **0.0001** |
| Pneumonia (viral) | 391,381 (0.1%) | ≤10 (0.9%) | **11.38 [2.90-44.72]** | **0.001** | **3.83 [1.01-14.67]** | **0.049** |
| **Renal** |  |  |  |  |  |  |
| Acute Renal Failure | 36,439,508 (7.8%) | 106 (10.4%) | 1.44 [0.95-2.20] | 0.089 | **2.65 [1.64-4.26]** | **<0.0001** |
| Hypocalcemia | 2,930,458 (0.6%) | 24 (2.4%) | **4.15 [1.79-9.61]** | **0.0009** | **5.17 [2.15-12.41]** | **0.0002** |
| Hyposmolality / Hyponatremia | 24,089,554 (5.2%) | 145 (14.3%) | **3.08 [2.09-4.54]** | **<0.0001** | **5.03 [3.34-7.59]** | **<0.0001** |
| Urinary Tract Infection | 38,469,041 (8.2%) | 82 (8.1%) | 0.78 [0.38-1.58] | 0.490 | **2.27 [1.34-3.84]** | **0.002** |

**Hospital course, disposition and mortality**

The mean [95% CI] LOS was significantly higher in patients with vs. without measles (4.8 [4.4-5.4] vs. 4.5 [4.4-4.5], P<0.01). Mean LOS increased slightly over time (Fig 2A) and was generally higher in older patients (Fig 2B). The strongest association with increased LOS was ≥2 chronic conditions (linear regression; adjusted beta [95% CI]: 0.76 [0.46-1.06], P<0.0001) (S2 Table).

**Fig 2.** Length of stay by year (A) and age (B) and cost of care by year (C) and age (D) for hospitalized patients with and without measles.

Inpatients with measles most frequently were admitted from the emergency room (51.6% [39.7-63.6%]) or referral from a physician, outpatient center or clinic (41.4% [31.2-55.6%]). When stratified by race/ethnicity, a lower proportion of whites vs. non-whites were admitted from the emergency room (white: 50.2%; black: 100.0%; Hispanic: 66.7%; Asian/Pacific Islander: 61.7%), while a higher proportion of whites vs. non-whites were admitted from referral from a physician, outpatient center or clinic (white: 49.8%; black: 0.0%; Hispanic: 33.3%; Asian/Pacific Islander: 38.3%). When stratified by age, admission from the ED was generally higher with increasing age (0-9: 40.2%; 10-19: 50.0%; 20-39: 79.8%; ≥40: 51.7%) and admission from referral by a physician, outpatient center or clinic was generally lower with increasing age (0-9: 55.7%; 10-19: 0.0%; 20-39: 20.2%; ≥40: 44.0%).

Inpatients with measles were most frequently routinely discharged to home or other self-care (84.3% [79.3-89.2%]), followed by transfer to other facilities (e.g. skilled nursing facilities, immediate care facilities) (8.5% [4.7-12.3%]). Inpatient mortality was not significantly higher in those with vs. without measles (proportion ± SEM: 3.3±1.2% vs. 2.3±0.01%, P=0.333).

**Cost**

The mean [95% CI] inflation-adjusted cost of inpatient care was not significantly different for those with vs. without a diagnosis of measles $7,438 [$6,446-$ 8,582] vs. $7,854 [$7,774-$7,935], P>0.05) and increased over time (Fig 2C). Mean costs generally increased with age in inpatients with and without measles (Fig 2D). The annual mean cost of measles hospitalization was $1,131,586 (range: $207,249 -$3,444,708) resulting in a total cost of $16,973,795 [$11,628,652-$22,318,934] from 2002-2016. Positive predictors of cost included ≥2 chronic conditions (linear regression; adjusted beta [95% CI], P-value; 0.73 [0.34, 1.12], P=0.0003) and increasing length of stay (2 days: 0.58 [0.01-1.14], P=0.046; ≥3 days: 1.23 [0.72-1.74], P<0.0001), while negative predictors of cost included decreasing urban population (not metropolitan or micropolitan: -1.11 [-2.07, -0.15], P=0.021) (S3 Table). **Discussion**

This study found increasing hospitalizations for measles in the US between 2002-2016, with prolonged and costly hospitalizations. The estimated total number of measles hospitalizations in the US from 1977-1984 was 13,710 [26], which increased from 1985-1996 to 28,047, driven largely by the 1989-1991 epidemic [27]. From 1996 to 2002 measles hospitalizations were at an all time low in US history with an estimated ≤23 cases annually [27]. While more recent data is lacking, an analysis of measles hospitalizations in a single children’s hospital in Minnesota from 2011-2017 showed 33 total cases, driven largely by a 2017 outbreak in the Somali-American community [30]. Despite measles being officially declared eliminated from the US in 2000 [23,24], this study found 1,018 weighted hospitalizations for measles (mean of 68 cases per year) and rising number of measles hospitalizations from 2002-2016.

Increasing measles hospitalization mirrors a resurgence in measles cases nationwide. The frequency of documented measles cases from 2001-2015 was estimated to be 1,789, resulting in a low (<1 per million persons) but climbing incidence over time [31]. Based on official case reporting to the CDC and its National Notifiable Diseases Surveillance System (NNDSS), from 2001-2008 there were 557 cases of measles resulting in an estimated 126 hospitalizations, which increased in 2009-2014 with 1,264 cases and an estimated 211 hospitalizations [32]. From 2002-2016, publically available data from NNDSS showed a total of 1,985 confirmed (i.e., laboratory-confirmed or epidemiologically linked cases) measles cases nationwide. Our analysis of the NIS during the same time period showed 1,014 weighted hospitalizations – a higher proportion of hospitalizations to cases than the CDC has estimated based on historical data (1 in 4 cases). The NNDSS is a passive reporting system that receives voluntary reports from state public health departments. Whereas, the NIS relies on discharge diagnoses coded as ICD-9 and ICD-10 data. Due to the passive nature of NNDSS reporting, previous studies suggested that reporting may be incomplete [32], particularly with hospitalized patients, as has been observed in past outbreaks [33]. Similar findings were seen internationally with other passive reporting systems [34]. Conversely, weighted discharge diagnoses using ICD-9 and ICD-10 codes may slightly overestimate measles cases in inpatients, as some cases with a measles diagnosis may have been rule-out diagnoses or misdiagnoses. Though, this is less likely since they are diagnoses provided at discharge that report the final diagnoses of those hospitalizations. Regardless, our results suggest there is increasing hospitalization for measles in the US over time.

A common finding in post-elimination years was that >80% of cases were in persons who were unvaccinated or had unknown vaccination status, and >60% of cases with philosophical or religious objection to vaccination [32], a theme echoed in other studies [35,36]. Decreasing incidence with age and decreasing proportion of imported and vaccinated cases suggests failure to vaccinate, rather than vaccine failure, was the driving force for the rising cases of measles in the US [31].

Measles can be perceived as a benign illness with no major consequences. However, as measles cases reemerge in the US, so do disease complications requiring hospitalization. We found gastrointestinal, hematologic, infectious, neurologic, ophthalmologic, pulmonary, and renal complications in inpatients with measles. From 1977-1984, the most common complications among hospitalized patients included pneumonia or other respiratory complications (34%), otitis media (8.5%), and encephalitis/convulsions/coma (3.4%); while pneumonia and otitis media frequency decreased with age, neurologic complications increased [26]. From 1987-2000, among all cases of measles, diarrhea (8.2%), otitis media (7.3%), pneumonia (5.9%), and encephalitis (0.1%) were the most prevalent [37]. From 2009-2014 complications were seen in 9% of cases, most commonly diarrhea (38%), pneumonia (33%), dehydration (25%), otitis media (16%), thrombocytopenia (10%), encephalitis (3%), pancytopenia (1%), and hepatitis (1%) [32]. We similarly found increased frequencies of these conditions, with dehydration, hyponatremia, pneumonia (primary or secondary bacterial/viral), acute renal failure, diarrhea, and thrombocytopenia showing highest prevalence among inpatients. Interestingly, keratitis was extremely rare. The rarity of keratoconjunctivitis may be explained by low rates of vitamin A deficiency in the US. Encephalitis showed the strongest positive association for measles diagnosis, with nearly 40-fold increased odds. Neurologic complications are associated with the most chronic morbidity. Post-measles encephalomyelitis can occur in 1 per 1,000 cases. Less commonly, measles inclusion body encephalitis or subacute sclerosing panencephalitis can present years after acute measles infection [22]. Previous studies showed that risk factors for severe illness include age <5 years and adults >20 years, immunodeficiency, pregnancy, malnutrition, crowding, and vitamin A deficiency [38].

Inpatient mortality was 3.3% (34 deaths) among measles inpatients, which was numerically higher than other inpatients (2.3%) and nearly 10-fold higher than previous mortality estimates of all cases of measles in the US (1 in 1,000). Hospitalized patients likely had more severe measles with complications and worse outcomes. The inpatient deaths observed in NIS are higher than reports of either no verified deaths from measles in NNDSS from 2009-2014 or one verified measles-related death in 1993-2002 from state death certificate data and the National Immunization Project that relies on direct reporting of states to the CDC [39]. Each of these reporting systems is limited by guidelines for measles case reporting (e.g. laboratory confirmation for CDC reporting), coding of death certificate diagnoses (limited to an acute and/or single direct cause of death), and state to federal communication. These limitations may result in underestimates of the true mortality of measles in the US [39].

Measles hospitalizations were more likely in non-white race/ethnicity, including Asians/Pacific Islanders, Hispanics, and Native American/others. Racial/ethnic disparities in measles vaccine coverage were thought to be reduced and/or potentially eliminated following targeted interventions by the CDC after the US measles epidemic of 1989-1991 [40]. However, there may be ongoing racial/ethnic differences in access to care, health literacy, and socioeconomic status driving the increased hospitalization observed in this study. This is supported by the finding that Asians/Pacific Islanders and Hispanics who were hospitalized with measles were more likely to present to the ED than whites. Hospitalization for measles was inversely associated with increasing age, which mirrored ours and others findings of decreased incidence with increased age [31]. While measles is thought to affect males and females similarly, our NIS data and nationwide CDC measles prevalence data from 2001-2015 (52.9% male) suggest a slight male predilection in the US [31]. While some have observed higher complication and mortality rates in females vs. males [41], others have found no significant difference [42].

The average cost per measles-related hospitalization was $7,438 with total cost from 2002-2016 of $16,973,795. There was an estimated $10,000 cost per hospitalization during the 1989-1991 US measles outbreak [11], and while recent cost estimates are limited, the median cost per measles hospitalization in a single children’s hospital from 2011-2017 was $5,291 [30]. Measles hospitalization costs include infection control required to stop nosocomial spread, such as isolation precautions, testing of exposed individuals, vaccine/immunoglobulin administration, tracing of cases, and the personnel hours required to carry out these tasks [43,44]. These represent the direct costs incurred in the inpatient setting, but do not capture indirect patient- and family-costs of measles hospitalizations. These costs also do not reflect the broader public health costs of measles, related to thorough case investigation, tracing of contacts to ascertain an index case, communication with the public, coordination between local, state, and federal health organizations, isolation and testing of exposed individuals, and post-exposure prophylaxis, which have been estimated to cost as high as $181,679 [45,46]. The public health cost of 16 measles outbreaks with 107 confirmed cases in the US in 2011 was estimated to be $2.7-$5.3 million [47]. Vaccination is both the most effective and cost-effective solution to prevent the spread of measles [48].

Study strengths include analysis of a nationally representative sample of all-payer data over a period of 15 years with over 96 million records. These results depict the serious and debilitating complications of measles infections resulting in hospitalization. However, milder measles cases are likely not captured in NIS. Limitations of this study include the lack of data on vaccination, serology or severity for measles, or inpatient treatment. Rare encephalitic complications can present years after measles and may be difficult to dissociate from more acute measles presentations. Identification of measles was performed using ICD-9-CM and ICD-10-CM codes and not verified by review of the health record or cross-referencing with measles registries. This could result in overestimation of the frequency and mortality rate of measles hospitalizations compared to expected estimates. However, recent studies suggest that ICD codes are effective in capturing several reportable diseases including measles and have improved over time [49].

The measles vaccine is inexpensive, extremely effective, and potentially lifesaving. The CDC estimates that among US children born from 1994–2013, the measles vaccine will prevent 322 million cases, 21 million hospitalizations, and 732,000 deaths over the course of their lifetimes, resulting in a savings of $295 billion in direct costs and $1.38 trillion in societal costs. More research and nationwide vaccination campaigns are needed to address measles resurgence in the US.

**References**

1. Gavish D, Kleinman Y, Morag A, Chajek-Shaul T (1983) Hepatitis and jaundice associated with measles in young adults. An analysis of 65 cases. Archives of internal medicine 143: 674-677.

2. Varavithya W, Aswasuwana S, Phuapradit P, Louisirirotchanakul S, Supavej S, et al. (1989) Etiology of diarrhea in measles. Journal of the Medical Association of Thailand = Chotmaihet thangphaet 72 Suppl 1: 151-154.

3. Wong RD, Goetz MB (1993) Clinical and laboratory features of measles in hospitalized adults. The American journal of medicine 95: 377-383.

4. Halsey NA, Modlin JF, Jabbour JT, Dubey L, Eddins DL, et al. (1980) Risk factors in subacute sclerosing panencephalitis: a case-control study. American journal of epidemiology 111: 415-424.

5. Detels R, Brody JA, McNew J, Edgar AH (1973) Further epidemiological studies of subacute sclerosing panencephalitis. Lancet 2: 11-14.

6. GIBBONS JL, MILLER HG, STANTON JB (1956) Para-infectious encephalomyelitis and related syndromes; a critical review of the neurological complications of certain specific fevers. The Quarterly journal of medicine 25: 427-505.

7. Aicardi J, Goutieres F, Arsenio-Nunes ML, Lebon P (1977) Acute measles encephalitis in children with immunosuppression. Pediatrics 59: 232-239.

8. MILLER DL (1964) FREQUENCY OF COMPLICATIONS OF MEASLES, 1963. REPORT ON A NATIONAL INQUIRY BY THE PUBLIC HEALTH LABORATORY SERVICE IN COLLABORATION WITH THE SOCIETY OF MEDICAL OFFICERS OF HEALTH. British medical journal 2: 75-78.

9. Makhene MK, Diaz PS (1993) Clinical presentations and complications of suspected measles in hospitalized children. The Pediatric infectious disease journal 12: 836-840.

10. Henneman PL, Birnbaumer DM, Cairns CB (1995) Measles pneumonitis. Annals of emergency medicine 26: 278-282.

11. Mason WH, Ross LA, Lanson J, Wright HT (1993) Epidemic measles in the postvaccine era: evaluation of epidemiology, clinical presentation and complications during an urban outbreak. The Pediatric infectious disease journal 12: 42-48.

12. Hussey GD, Clements CJ (1996) Clinical problems in measles case management. Annals of tropical paediatrics 16: 307-317.

13. Markowitz LE, Nieburg P (1991) The burden of acute respiratory infection due to measles in developing countries and the potential impact of measles vaccine. Reviews of infectious diseases 13 Suppl 6: S555-561.

14. Sommer A (1990) Xerophthalmia, keratomalacia and nutritional blindness. International ophthalmology 14: 195-199.

15. Foster A, Sommer A (1986) Childhood blindness from corneal ulceration in Africa: causes, prevention, and treatment. Bulletin of the World Health Organization 64: 619-623.

16. Foster A, Sommer A (1987) Corneal ulceration, measles, and childhood blindness in Tanzania. The British journal of ophthalmology 71: 331-343.

17. HUDSON JB, WEINSTEIN L, CHANG TW (1956) Thrombocytopenic purpura in measles. The Journal of pediatrics 48: 48-56.

18. Wairagkar NS, Gandhi BV, Katrak SM, Shaikh NJ, Parikh PR, et al. (1999) Acute renal failure with neurological involvement in adults associated with measles virus isolation. Lancet 354: 992-995.

19. Kambarami RA, Nathoo KJ, Nkrumah FK, Pirie DJ (1991) Measles epidemic in Harare, Zimbabwe, despite high measles immunization coverage rates. Bulletin of the World Health Organization 69: 213-219.

20. Goodson JL, Seward JF (2015) Measles 50 Years After Use of Measles Vaccine. Infectious disease clinics of North America 29: 725-743.

21. Clemmons NS, Gastañaduy PA, Fiebelkorn AP, Redd SB, Wallace GS, et al. (2015) Measles - United States, January 4-April 2, 2015. MMWR Morbidity and mortality weekly report 64: 373-376.

22. Moss WJ, Griffin DE (2012) Measles. Lancet 379: 153-164.

23. Papania MJ, Wallace GS, Rota PA, Icenogle JP, Fiebelkorn AP, et al. (2014) Elimination of endemic measles, rubella, and congenital rubella syndrome from the Western hemisphere: the US experience. JAMA pediatrics 168: 148-155.

24. Katz SL, Hinman AR (2004) Summary and conclusions: measles elimination meeting, 16-17 March 2000. The Journal of infectious diseases 189 Suppl 1: S43-47.

25. Paules CI, Marston HD, Fauci AS (2019) Measles in 2019 - Going Backward. The New England journal of medicine: NEJMp1905099.

26. Markowitz LE, Tomasi A, Sirotkin BI, Carr RW, Davis RM, et al. (1987) Measles hospitalizations, United States, 1977-84: comparison with national surveillance data. American journal of public health 77: 866-868.

27. Lee B, Ying M, Papania MJ, Stevenson J, Seward JF, et al. (2004) Measles hospitalizations, United States, 1985-2002. The Journal of infectious diseases 189 Suppl 1: S210-215.

28. Zhou F, Reef S, Massoudi M, Papania MJ, Yusuf HR, et al. (2004) An economic analysis of the current universal 2-dose measles-mumps-rubella vaccination program in the United States. The Journal of infectious diseases 189 Suppl 1: S131-145.

29. Benjamini Y, Hochberg Y (2018) Controlling the False Discovery Rate: A Practical and Powerful Approach to Multiple Testing. Journal of the Royal Statistical Society: Series B (Methodological) 57: 289-300.

30. Hester G, Nickel A, LeBlanc J, Carlson R, Spaulding AB, et al. (2019) Measles Hospitalizations at a United States Children&apos;s Hospital 2011-2017. The Pediatric infectious disease journal 38: 547-552.

31. Clemmons NS, Wallace GS, Patel M, Gastañaduy PA (2017) Incidence of Measles in the United States, 2001-2015. JAMA 318: 1279-1281.

32. Fiebelkorn AP, Redd SB, Gastañaduy PA, Clemmons N, Rota PA, et al. (2017) A Comparison of Postelimination Measles Epidemiology in the United States, 2009–2014 Versus 2001–2008. Journal of the Pediatric Infectious Diseases Society 6: 40-48.

33. Davis SF, Strebel PM, Atkinson WL, Markowitz LE, Sutter RW, et al. (1993) Reporting efficiency during a measles outbreak in New York City, 1991. American journal of public health 83: 1011-1015.

34. Van Den Hof S, Smit C, Van Steenbergen JE, De Melker HE (2002) Hospitalizations during a measles epidemic in the Netherlands, 1999 to 2000. The Pediatric infectious disease journal 21: 1146-1150.

35. Parker AA, Staggs W, Dayan GH, Ortega-Sanchez IR, Rota PA, et al. (2006) Implications of a 2005 measles outbreak in Indiana for sustained elimination of measles in the United States. The New England journal of medicine 355: 447-455.

36. Phadke VK, Bednarczyk RA, Salmon DA, Omer SB (2016) Association Between Vaccine Refusal and Vaccine-Preventable Diseases in the United States: A Review of Measles and Pertussis. JAMA 315: 1149-1158.

37. Perry RT, Halsey NA (2004) The clinical significance of measles: a review. The Journal of infectious diseases 189 Suppl 1: S4-16.

38. Butler JC, Havens PL, Sowell AL, Huff DL, Peterson DE, et al. (1993) Measles severity and serum retinol (vitamin A) concentration among children in the United States. Pediatrics 91: 1176-1181.

39. Gindler J, Tinker S, Markowitz L, Atkinson W, Dales L, et al. (2004) Acute measles mortality in the United States, 1987-2002. The Journal of infectious diseases 189 Suppl 1: S69-77.

40. Walker AT, Smith PJ, Kolasa M, (CDC) CfDCaP (2014) Reduction of racial/ethnic disparities in vaccination coverage, 1995-2011. MMWR supplements 63: 7-12.

41. Organization WH (2007) Addressing sex and gender in epidemic-prone infectious diseases.

42. Barkin RM (1975) Measles mortality: a retrospective look at the vaccine era. American journal of epidemiology 102: 341-349.

43. Wendorf KA, Kay M, Ortega-Sanchez IR, Munn M, Duchin J (2015) Cost of measles containment in an ambulatory pediatric clinic. The Pediatric infectious disease journal 34: 589-593.

44. Stuart RL, Bradford J, Leszkiewicz P, Wilson J, Gillespie EE (2010) The costs of containing measles within a health care service. Healthcare infection 15: 43-46.

45. Dayan GH, Ortega-Sanchez IR, LeBaron CW, Quinlisk MP, Team IMR (2005) The cost of containing one case of measles: the economic impact on the public health infrastructure--Iowa, 2004. Pediatrics 116: e1-4.

46. Marx GE, Chase J, Jasperse J, Stinson K, McDonald CE, et al. (2017) Public Health Economic Burden Associated with Two Single Measles Case Investigations - Colorado, 2016-2017. MMWR Morbidity and mortality weekly report 66: 1272-1275.

47. Ortega-Sanchez IR, Vijayaraghavan M, Barskey AE, Wallace GS (2014) The economic burden of sixteen measles outbreaks on United States public health departments in 2011. Vaccine 32: 1311-1317.

48. Ghebrehewet S, Thorrington D, Farmer S, Kearney J, Blissett D, et al. (2016) The economic cost of measles: Healthcare, public health and societal costs of the 2012-13 outbreak in Merseyside, UK. Vaccine 34: 1823-1831.

49. Watzlaf VJM, Garvin JH, Moeini S, Anania-Firouzan P (2007) The effectiveness of ICD-10-CM in capturing public health diseases. Perspectives in health information management 4: 6.

**Acknowledgements**

None

**Supporting information**

**S1 Table. ICD-9-CM and ICD-10-CM Codes Used to Identify Measles and Comorbidities.**

**S2 Table. Predictors of Length of Stay in Patients with Measles.**

**S3 Table. Predictors of Cost of Care in Patients with Measles.**
